# Self-Assembled Al Nanostructure/ZnO Quantum Dot Heterostructures for High Responsivity and Fast UV Photodetector

**DOI:** 10.1007/s40820-020-00455-9

**Published:** 2020-05-22

**Authors:** Sisi Liu, Ming-Yu Li, Jianbing Zhang, Dong Su, Zhen Huang, Sundar Kunwar, Jihoon Lee

**Affiliations:** 1 School of Optical and Electronic Information, Huazhong University of Science and Technology, Wuhan, 430074 People’s Republic of China; 2 School of Science, Wuhan University of Technology, Wuhan, 430070 People’s Republic of China; 3 Wuhan National Laboratory for Optoelectronics (WNLO) and School of Engineering Sciences, Huazhong University of Science and Technology, Wuhan, 430074 People’s Republic of China; 4 College of Electonics and Information, Kwangwoon University, Nowon-gu, Seoul, 01897 Republic of Korea; 5 Institute of Nanoscale Science and Engineering, University of Arkansas, Fayetteville, AR 72701 USA

**Keywords:** Al/ZnO heterostructure photodetectors, Plasmonic enhancement, ZnO quantum dots, Self-assembled Al nanostructures

## Abstract

**Highlights:**

- High performance Al nanostructures/ZnO quantum dots heterostructure photodetectors with a controllable geometry of the Al nanostructures are demonstrated.
- Light utilization of the photoactive layers is significantly boosted with the Al nanostructures.
- The light confinement effect is inherently determined by the geometries of the Al nanostructures.

**Abstract:**

Light confinement induced by spontaneous near-surface resonance is inherently determined by the location and geometry of metallic nanostructures (NSs), offering a facile and effective approach to break through the limitation of the light-mater interaction within the photoactive layers. Here, we demonstrate high-performance Al NS/ZnO quantum dots (Al/ZnO) heterostructure UV photodetectors with controllable morphologies of the self-assembled Al NSs. The Al/ZnO heterostructures exhibit a superior light utilization than the ZnO/Al heterostructures, and a strong morphological dependence of the Al NSs on the optical properties of the heterostructures. The inter-diffusion of Al atoms into ZnO matrixes is of a great benefit for the carrier transportation. Consequently, the optimal photocurrent of the Al/ZnO heterostructure photodetectors is significantly increased by 275 times to ~ 1.065 mA compared to that of the pristine ZnO device, and an outstanding photoresponsivity of 11.98 A W^−1^ is correspondingly achieved under 6.9 mW cm^−2^ UV light illumination at 10 V bias. In addition, a relatively fast response is similarly witnessed with the Al/ZnO devices, paving a path to fabricate the high-performance UV photodetectors for applications.
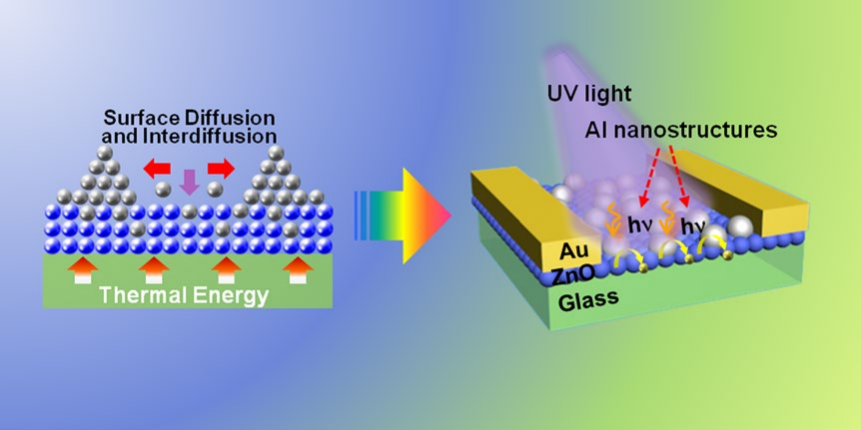

**Electronic supplementary material:**

The online version of this article (10.1007/s40820-020-00455-9) contains supplementary material, which is available to authorized users.

## Introduction

Owing to their excellent characteristics of intrinsic anti-interference from the visible region [1], filter free [2], and high radiation hardness [3], UV photodetectors (PDs) have been widely witnessed in civilian and military fields, including intersatellite communications [4], optical imaging [5], flame sensing [6], environmental monitoring [7, 8], and missile detection [9]. Among various UV photo-electronic materials (i.e., GaN, SiC, SnO_2_, and TiO_2_ [10, 11]), ZnO has great potential to fabricate high-performance UV photodetectors because of its wide direct bandgap of ~ 3.37 eV, high saturated carrier drift rate, large exciton energy (60 meV) [12, 13]. Whereas, the conventional ZnO single crystal and thin-film photodetectors commonly suffer from a large dislocation density, excessive grain boundaries, and high-cost fabrication, resulting in radical deterioration of photoresponse for the devices [14]. Meanwhile, the solution-processed ZnO nanostructures (NSs) (i.e., quantum dots (QDs), nanowires, and nanodisks) emerged with high adjustability, physical flexibility, and low cost, offering a promising approach to achieve the high-performance detectors in practical fabrication [10]. Additionally, the high specific surface area of the ZnO NSs allows additional oxygen absorption, and a relatively lower dark current and high on/off ratio can correspondingly be expected with extended low-conductive depletion regions [10, 15]. However, the dimension of the ZnO NSs inevitably limits the light utilization and carrier transportation, which have become a huge challenge for the fabrication of ultra-sensitive photodetectors working with a fast response [15].

Localized surface plasmon resonance (LSPR), spontaneously collective charge oscillation on the surface of metallic NSs [16], provides a facile way to boost the light absorption for photoactive layers via concentrating the incident lights at the metal NSs-semiconductor interface [17]. The resonance wavelength can be inherently varied depending on metals, and the traditional noble metals (Au and Ag) exhibit a radical response in the visible region [18]. Thus, cost-effective metallic NSs with a desired response wavelength are required to effectively intensify the light utilization of ZnO layers. According to the Mie theory, the dielectric function of Al possess a negative real part (*ε*′) and relatively low imaginary part (*ε*″) throughout the whole region [19], suggesting an excellent potential to guarantee a superior plasmon resonance within the response wavelength range of ZnO photodetectors. In addition, Al is regarded as earth-abundant and low-cost, and Al atoms can easily diffuse into ZnO matrixes under high temperature annealing to form a layer with a large carrier mobility [20]. In the light of the advantages, Al NSs potentially provides an optimized solution for the fabrication of high-performance ZnO UV photodetectors. Up to date, researchers have focused on the fabrication of Al-doped ZnO (AZO) transparent conductive oxide thin films, and the application of Al NSs in ZnO photodetectors has been rarely reported.

Herein, we report a high-performance ZnO QD UV photodetector with the self-assembled Al NSs fabricated via the cost-effective and facile solid-state dewetting method. The solar energy utilization of the photoactive layers strongly depends on the configuration of the heterostructures and morphologies of the resulting Al NSs due to the variation of the plasmonic coupling. As a result, the UV photodetector with Al/ZnO heterostructures fabricated at 500 °C exhibit an excellent photocurrent of 1.065 mA and photoresponsivity of 11.98 A W^−1^ under 6.9 mW cm^−2^ light illumination at a bias of 10 V. In addition, the fast response speed is witnessed with each device, and the *τ*_rise_ and *τ*_decay_ of the Al/ZnO heterostructure photodetector even decrease to ~ 0.79 s and ~ 0.24 s, suggesting a promising solution for the high-performance photodetector fabrication in the practical applications.

## Experimental

### ZnO QDs Synthesis

ZnO QDs were synthesized by the solvothermal method as reported in our previous work [1, 21]. In brief, 2.49 g zinc acetate (Zn(CH_3_COO)_2_, 99.99%, Aladdin, CAS: 5970-45-6) was dissolved into 126 mL methanol by stirring at 60 °C. Subsequently, 23 ml potassium hydroxide (KOH) methanol solution (CAS: 67-56-1) by dissolving 1.29 g KOH (85%, Aladdin, CAS: 1310-58-3) slowly dropped into the zinc acetate methanol solution. The mixtures were heating at 60 °C for 2.25 h with vigorously stirring, and then were naturally cooled to room temperature. After twice washing with methanol by precipitation method, the product was finally dispersed in a mixed solvent of chloroform and methanol (6 mL, 2:1 by volume). As revealed in Fig. S1a, the resulting ZnO QDs have average size of 7 nm, and the absorption bands well developed below 360 nm in the absorbance spectra in Fig. S1b.

### Heterostructure Photodetector Fabrication

**Fig. 1:**
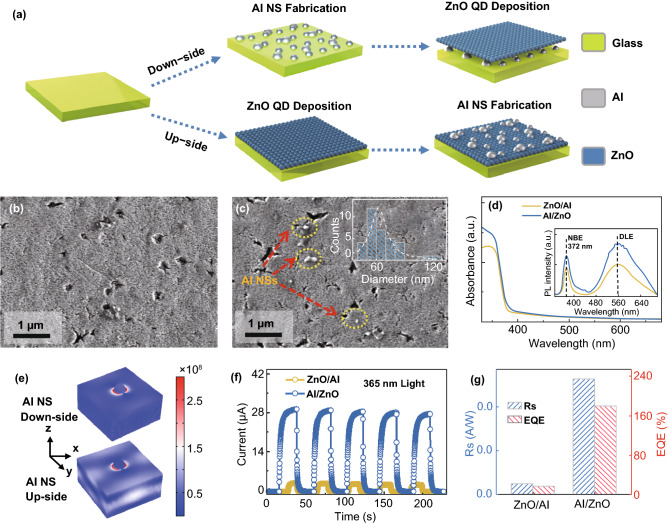
**a** Schematics of the fabrication process for heterostructure photodetectors with the assembled Al nanostructures under (ZnO/Al sample) and on (Al/ZnO sample) the ZnO photoactive layer. SEM images of the **b** ZnO/Al and **c** Al/ZnO devices. (Insets) The size distribution histograms of the Al NSs. **d** Absorbance spectra and room-temperature PL emission spectra (insets) of the Al/ZnO and ZnO/Al devices. **e** Electromagnetic (EM) filed distribution of the samples Al/ZnO and ZnO/Al via the COMSOL simulation. **f** Temporal photoresponse of the Al/ZnO and ZnO/Al devices under 365 nm light illumination (6.9 mW cm^−2^) at a bias of 10 V. **g** Photoresponsivity (*R*_*s*_) and external quantum efficiency (EQE) of each device

The photodetectors were fabricated on the glass substrates with two configurations to study the influence of stacking-order between ZnO QD and Al NSs on the light trapping as shown in Fig. 1a. Prior to the fabrication, the glass substrates were successively cleaned through 15 min ultrasonification in deionized water and acetone, and then were degassed at 500 °C for 30 min under a vacuum below 1 × 10^−4^ Torr to remove the residual chemicals on the surface. After cleaning, Al thin films with a thickness of 6 nm was deposited on the glass via thermal evaporation at a rate of 0.1 nm s^−1^ under 1 × 10^−4^ Pa. The self-assembled Al NSs were fabricated via the solid-state dewetting method [22] at 500 °C in a rapid thermal annealing furnace (OTF-1200-4-RTP, Hefeikejing Materials Technology Co., Ltd., China) under 1 × 10^−5^ Torr (a molecular pump was equiped to extend the pressure range). As shown in Fig. S1c, the pronounced absorption bands of the resulting Al NSs appeared below 400 nm, which exactly matched the response wavelength range of ZnO QDs. The ZnO QD photoactive layers were subsequently deposited on the Al nanostructures by layer-by-layer spin-coating at a speed of 2000 rpm for 30 s (the ZnO/Al heterostructure photodetector). To evaluate the temperature effects, the Al NSs was fabricated at 300 °C (Al/ZnO-300), 400 °C (Al/ZnO-400), 500 °C (Al/ZnO-500) with an identical deposition thickness of 6 nm on the spin-coated ZnO QD thin films. To investigate the deposition thickness effects, the Al NSs were fabricated on ZnO QD thin films at a fixed annealing temperature of 500 °C with various deposition thicknesses: 6 nm (Al/ZnO-6 nm), 8 nm (Al/ZnO-8 nm), and 10 nm (Al/ZnO-10 nm). As reference, the pristine ZnO devices were annealed at 300 to 500 °C, and the device structure is shown in Fig. S1d. Finally, a pair of Au electrodes with a length of 4 mm and a gap distance of 200 μm was deposited on each sample to fabricate photodetector as shown in Fig. S2a. The Commercial COMSOL solution software was employed for the simulation of the EM field distribution of the heterostructures, and the parameters in simulations were the statistic values required from the SEM images.

### Characterization

The absorption spectra of the Al NSs and Al/ZnO heterostructures were recorded with a UV–vis spectrophotometer (UV 3600 Plus, Japan). The room-temperature photoluminescence (PL) spectra were obtained via a Raman microscope with an excitation laser of 325 nm (LabRAM HR800, Horiba JobinYvon Corp., France). The transmission electron microscope (TEM, TF20, FEI Tecnai Corp., USA) was utilized for morphological characterization of the ZnO QD. The scanning electron microscope (SEM, GeminiSEM 300, Carl Zeiss Microscopy GmbH, Corp., Germany) was employed for the morphological characterization of the Al/ZnO heterostructures. The element analysis was carried out with the energy dispersive spectroscopy (EDS, GeminiSEM 300, Carl Zeiss Microscopy GmbH, Corp., Germany) and X-ray photoelectron spectroscopy using Al Kα excitation (XPS, Thermo Fisher, EscaLab 250Xi). The performance of the photodetectors was measured by a semiconductor device analyzer (Agilent technologies B1500A, America) inside an optically and electrically sealed box. The lighting source was light-emitting diodes controlled by a functional generator (Agilent 33210A).

## Results and Discussion

### Configuration Effect on Device Performance

Figure 1 shows the configuration effect on the performance of ZnO QD photodetectors with the self-assembled Al NSs fabricated on (Al/ZnO)/under (ZnO/Al) photoactive layers, and the fabrication process is depicted in Fig. 1a. As shown in Fig. 1b, c, continuous geometry of the ZnO QD thin films was witnessed on each device, and sparse Al NSs spontaneously aggregated on the ZnO QD thin films with an average diameter (*D*_ave_) of ~ 64.8 nm for the Al/ZnO sample, suggesting a favorable surface diffusion of Al atoms after annealing. To understand the carrier generation process for each configuration, the absorption and room-temperature PL after 325 nm laser excitation were investigated as shown in Fig. 1d. The absorption edge peak below ~ 375 nm was similarly observed for each sample, and absorption was obviously enhanced for the Al/ZnO sample in the light of the concentrated incident lights with intensified near-surface EM fields. Correspondingly, the near-band-edge (NBE) excitonic emission peak at ~ 372 nm and defect-related emission (DLE) peak at ~ 560 nm were drastically increased for Al/ZnO sample than that of the ZnO/Al sample, which was because of the intensified recombination with extra electron–hole pairs excited by the concentrated incident lights [23]. As clearly shown in Fig. 1e, the EM in ZnO layers was drastically intensified for the Al/ZnO sample, indicating the light confinement effect for the absorption. In addition, the comparison of the performance of the Al/ZnO and ZnO/Al devices are shown in Figs. 1f–g and S2b, c. As shown in Fig. S2b, a slightly higher current (*I*_d_) of the Al/ZnO detector was similarly witnessed at each bias voltage in the dark, which was probably attributed to the increased conductivity of ZnO layers induced by the diffusion for Al atoms into ZnO lattice during annealing [24]. Meanwhile, the photocurrents (*I*_ph_) of the Al/ZnO detector were obviously higher than that of the ZnO/Al one with a variation of voltage bias due to the additional excited carriers by the concentrated lights as revealed in Fig. S2c. As a consequence, the *I*_ph_ of the Al/ZnO photodetector remarkably enhanced 10.3 times than that of the ZnO/Al one at a bias of 10 V as shown in Fig. 1f, and the temporal response in multiple on/off cycles verified the superior stability and repeatability of the resulting devices. The responsivity (*R*_*s*_) of detectors suggests of the electrical signal generation converting from incident lights, which can be expressed as Eq. 1 [25]:

1 $$R_{s} = \frac{{(I_{\text{ph}} - I_{\text{d}} )}}{{P_{0} A}}$$

where *P*_0_ is the light power (6.9 mW cm^−2^) and *A* is the active area of the devices. As shown in Fig. 1g, the Al/ZnO device exhibited the *R*_*s*_ of ~ 0.529 A W^−1^, which increased by 926% than the *R*_*s*_ of ZnO/Al device. The external quantum efficiency (EQE), the ability of electrons excitation by incident photons, can be given with Eq. 2 [26]:

2 $${\text{EQE}} = R_{s} \times \frac{1240}{\lambda } \times 100$$

where *λ* is the incident wavelength of 365 nm. As shown in Fig. 1g, the EQE increased by 10.3 times to ~ 179.6% for the Al/ZnO device in comparison with the ZnO/Al device.

### Annealing Temperature Effect on Device Performance

**Fig. 2:**
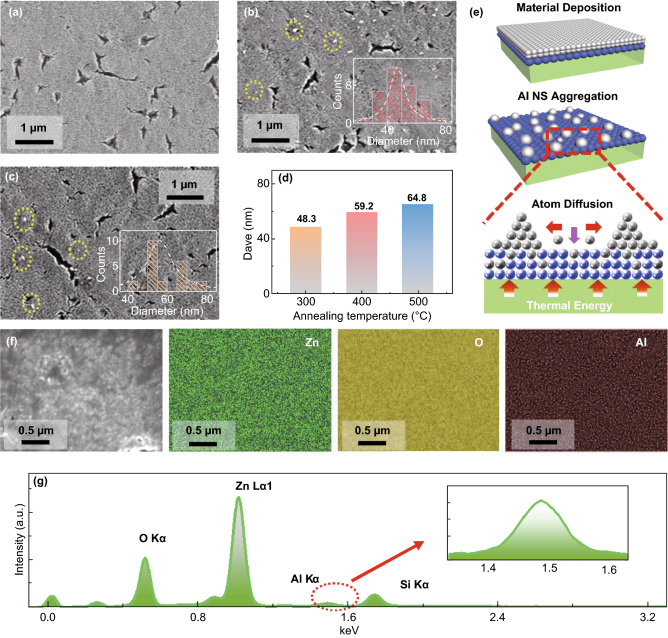
SEM images of the **a** pristine ZnO, Al/ZnO heterostructures fabricated at **b** 300 °C and **c** 400 °C. (Insets) The size distribution histograms of the resulting Al NSs. **d** Average diameters (*D*_ave_) of the Al NSs fabricated at various annealing temperatures. **e** Schematics of the fabrication of the Al NSs on ZnO QD thin films. **f** EDS maps of the heterostructures fabricated at 500 °C. **g** Corresponding EDS spectra

To further understand the evolution behavior, the self-assembled Al NSs were fabricated on ZnO thin films with a variation of annealing temperatures between 300 and 500 °C on ZnO QD thin films as shown in Fig. 2. As shown in Figs. 2a–c, the Al atoms spontaneously aggregated into the NSs at 300 °C when compared with the relatively smooth morphology of the pristine ZnO thin films, and the size expansion of the sparse Al NSs was observed as a function of annealing temperatures. Correspondingly, the *D*_*ave*_ of the resulting Al NSs gradually increased from ~ 48.3 to ~ 64.8 nm from 300 to 500 °C as plotted in Fig. 2d. Generally, the Al atoms were sensitive to thermal energy, and the competition between surface aggregation and inter-diffusion of Al atoms determined the resulting morphology of the Al NSs. As depicted in Fig. 2e, Al atoms were activated to randomly nucleate with thermal energy supply, and the diffusion length of Al atoms (*L*_Al_) on the ZnO thin films can be given with Eq. 3 [27, 28]

3 $$L_{\text{Al}} \propto \sqrt {\exp ( - E_{\text{Al}} /kT) \times t}$$

where *E*_Al_ is the activation energy, *k* is the Boltzmann constant, *T* is the annealing temperature, and *t* is the residence time of Al atoms on the ZnO thin films. Thus, the *L*_Al_ can be inherently determined by the *T*, and the size expansion of the Al NSs was correspondingly observed due to the favorable surface diffusion of atoms with a broadened *L*_Al_. Meanwhile, the inter-diffusion of Al atoms into ZnO matrixes was concurrently accelerated along with the increasing *T*, resulting in an evident density decrease of Al NSs during the evolution. Additionally, the element distribution of the Al/ZnO heterostructures fabricated at 500 °C was analyzed by EDS as shown in Fig. 2f, g. As observed in Fig. 2f, the Zn (green), O (yellow), and Al (red) uniformly distributed throughout the whole structures, indicating the inter-diffusion of Al atoms. The existence of Al can be further evidenced with the Kα peak at 1.49 eV as shown with the EDS spectra in Fig. 2g [29].

**Fig. 3:**
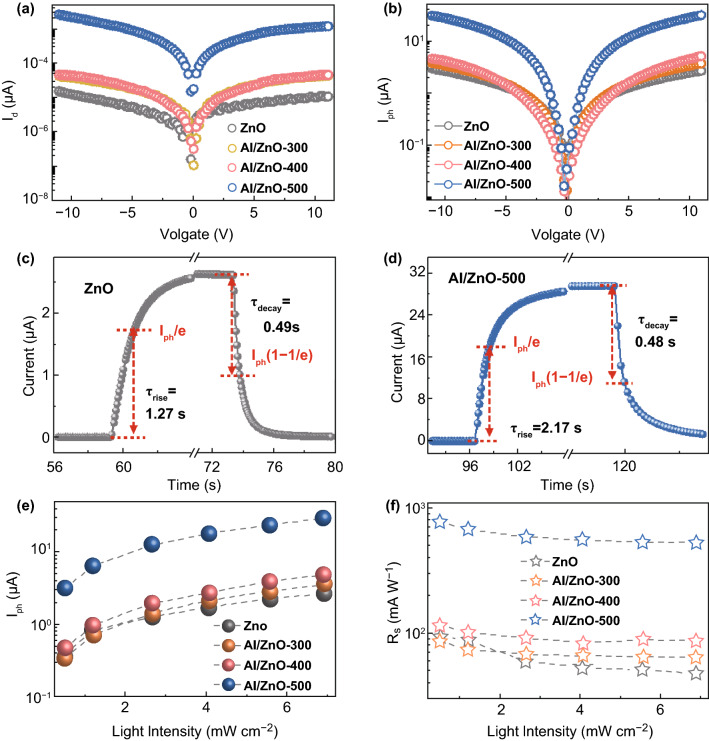
Photoresponse of the Al/ZnO heterostructure UV photodetectors fabricated at various temperatures. Current–voltage curves of each device **a** in the dark and **b** under 365 nm light illumination (6.9 mW cm^−2^) at a 10 V bias. Time-resolved photoresponse of the **c** pristine ZnO and **d** Al/ZnO devices under 365 nm light illumination at a 10 V bias. **e** Photocurrent and **f** photoresponsivity of each device under 365 nm UV illumination as a function of light intensities

The performance of the Al/ZnO heterostructure UV photodetectors with the self-assembled Al NSs fabricated at diverse annealing temperatures was systematically investigated as shown in Fig. 3, and the corresponding values are summarized in Table 1. As shown in Fig. 3a, the Al/ZnO heterostructure UV photodetectors exhibited relatively higher *I*_d_ than that of pristine one at each bias due to the introduction of Al atoms [24], and the *I*_d_ gradually increased as a function of the annealing temperature arising from the accelerated inter-diffusion of Al atoms. Meanwhile, the temperature dependence on the *I*_ph_ was observed at each bias, and the *I*_ph_ of the Al/ZnO detectors was obviously improved in comparison with the pristine ZnO device under 365 light illumination as revealed in Fig. 3b. As a result, the *I*_ph_ of the detector Al/ZnO-500 noticeably increased by 1010% to ~ 29.2 μA than the pristine ZnO device of ~ 2.63 μA at 10 V bias under the UV light illumination as summarized in Table 1. In addition, the *I*_ph_ of ZnO photodetectors significantly lower than that of Al/ZnO heterostructure device at each annealing temperature, as shown in Fig. S2d. Moreover, each Al/ZnO heterostructure photodetector sensitively responded to the UV light with a fast speed, which can be verified with the rise time (*τ*_rise_, the period of the current increased to *I*_ph_/e) and decay time (*τ*_decay_, period of the current decreased to *I*_ph_× (1 − 1/e)) as shown in Figs. 3c, d and **S3**. Compared with the pristine ZnO device, the *τ*_rise_ and *τ*_decay_ of the device Al/ZnO-300 decreased to ~ 0.79 s and ~ 0.24 s. The response speed of the Al/ZnO heterostructure photodetector was apparently superior to previously reported ZnO-based UV photodetectors [4, 30–32], and was even faster than our previously reported Cu/ZnO hybrid architecture photodetectors [1]. The light intensity dependence on the *I*_ph_ was compared for the Al/ZnO heterostructure photodetector at an identical bias of 10 V as shown in Fig. S4. Compared with the pristine ZnO photodetector, one order higher *I*_ph_ was constantly witnessed with the device Al/ZnO-500 with a variation of the light intensity from 0.5 to 6.9 mW cm^−2^ as revealed in Fig. 3e. Consequently, the *R*_*s*_ of the device Al/ZnO-500 significantly increased by 11 times to ~ 528.5 mA W^−1^ when compared with the pristine ZnO device at 6.9 mW cm^−2^, and it further increased to ~ 779.5 mA W^−1^ at 0.5 mW cm^−2^ as shown in Fig. 3f. Under the light intensity of 0.5 mW cm^−2^, the device Al/ZnO-500 also showed an excellent EQE of ~ 264.8%, which was about 8.3 times higher than that of the pristine ZnO detector as shown in Fig. S5a. As an aggregative indicator to evaluate the performance of a photodetector, the normalized detectivity (D*) can be expressed by Eq. 4 [12]:

4 $$D^{*} = \frac{{(I_{\text{ph}} - I_{\text{d}} )}}{{P_{0} \sqrt {2qI_{\text{d}} A} }}$$

where *q* is the elementary charge. Although the distinct increases in *I*_ph_ were achieved by introducing the Al NSs, the D* of the Al/ZnO heterostructure photodetectors slightly deteriorated due to the elevated *I*_d_ as shown in Fig. S5b. However, the D* of the device Al/ZnO-300 still maintained comparable with that of the pristine ZnO one, and it was still superior to the performance of ZnO photodetectors in previous works [33, 34].

**Table 1:** The dark current (*I*_d_), photocurrent (*I*_ph_), photoresponsivity (*R*_*s*_), and the external quantum efficiency (EQE) of the pristine ZnO and heterostructure photodetectors; the applied bias voltage was 10 V

Samples	*I*_d_ (nA)	*I*_ph_ (μA)	*R*_*s*_ (mA W^−1^)	EQE (%)
ZnO	0.039	2.63	47.64	16.19
ZnO/Al	0.03	2.84	51.49	17.49
Al/ZnO-300	0.11	3.54	64.13	21.79
Al/ZnO-400	2.57	4.79	86.67	29.45
Al/ZnO-500	26.1	29.20	528.51	179.55
Al/ZnO-8 nm	1.49 × 10^5^	768.29	11,220.91	3812.03
Al/ZnO-10 nm	4.04 × 10^5^	1065.2	11,976.45	4068.71

**Fig. 4:**
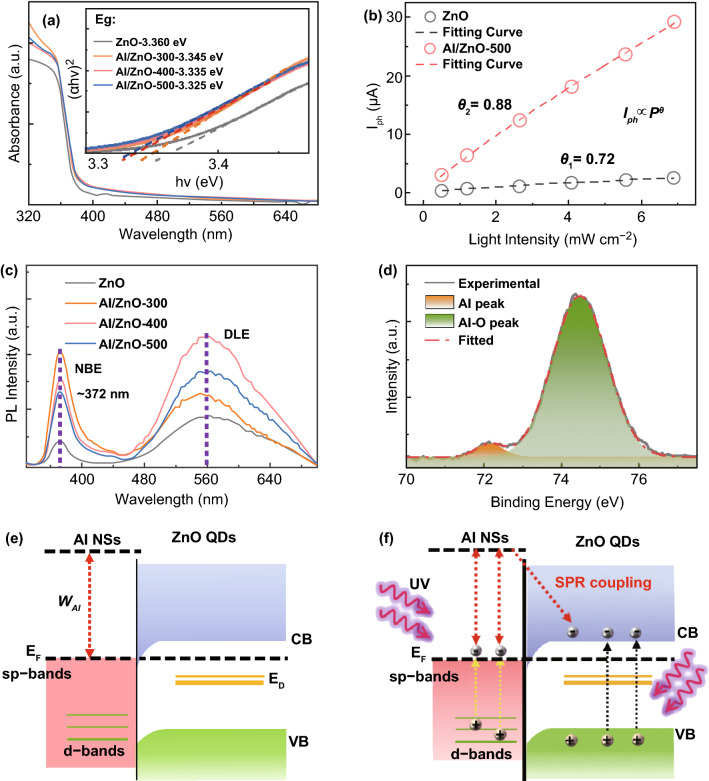
**a** Absorbance spectra of the Al/ZnO heterostructures fabricated at different temperatures. (Inset) Tauc plots of the heterostructures. **b** The light intensity dependence on photocurrent for the pristine ZnO and Al/ZnO photodetectors. **c** Room-temperature PL emission spectra of the photodetectors. **d** XPS spectra of Al 2p 3/2 signatures on the Al/ZnO-500. The band diagrams of Al/ZnO photodetectors **e** in the dark and **f** under UV light illumination

To ascertain the enhancement mechanism for the photodetectors, the light-mater interactions for the Al/ZnO heterostructure photodetectors were investigated as shown in Fig. 4. As revealed shown in Fig. 4a, the absorption edge peaks appeared below ~ 375 nm for each device, and the absorption peaks of the Al/ZnO heterostructures slightly increased as a result of the intensive light confinement with LSPR. Given that, the inter-diffusion of Al atoms can result in a strong electronegative mismatch between Zn and Al atoms and emergence of impurity energy level in the bandgap of ZnO [35], a gradual redshift of optical bandgap from 3.36 eV (the pristine ZnO) to 3.325 eV (the Al/ZnO-500 sample) was observed. As shown in Fig. 4b, the relationship between the light intensity and photocurrent for the pristine ZnO and Al/ZnO-500 devices was fitted by Eq. 5 [36]:

5 $$I_{\text{ph}} \propto P_{0}^{\theta }$$

where *θ* is the power law factor. The value of *θ* increased from 0.72 (pristine ZnO) to 0.88 (Al/ZnO-500), suggesting the generation of additional electron–hole pairs with the existence of the Al NSs [15]. Compared to pristine ZnO, the NBE peaks at ~ 372 nm and DLE peaks at ~ 560 nm were drastically increased for the Al/ZnO heterostructures as shown in Fig. 4c, which can be resulted from the intensified recombination of the excess carriers excited with the concentrated lights and the “hot electrons” injection from the Al NSs [37]. Whereas, the near-surface electromagnetic fields were gradually boosted by the Al NSs with a larger dimension at higher temperatures, inhibiting the recombination of the electron–hole pairs for the PL emission. Figure 4d shows the deconvolution of the Al 2p_3/2_ XPS peak of Al/ZnO-500 sample. The metallic Al peak at ~ 72.2 eV and oxidized Al peak at ~ 74.2 eV were simultaneously observed in the Gaussian fitted asymmetry peak, manifesting the co-existence of the Al NSs and the inter-diffusion of Al atoms [38]. In addition, the band diagrams of the Al NSs and ZnO QD in the dark and under UV light illumination is depicted in Fig. 4e, f. Given that ZnO nanostructures possess a higher work function of 5.2–5.3 eV than that of Al (~ 4.3 eV) [39], the downward band bending spontaneously formed as shown in Fig. 4e. As shown in Fig. 4f, the excited “hot electrons” from the Al NSs with the UV lights partially injected into the conductive band of ZnO, which further resulted in the increases of the *I*_ph_.

### Deposition Thickness Effect on Device Performance

**Fig. 5:**
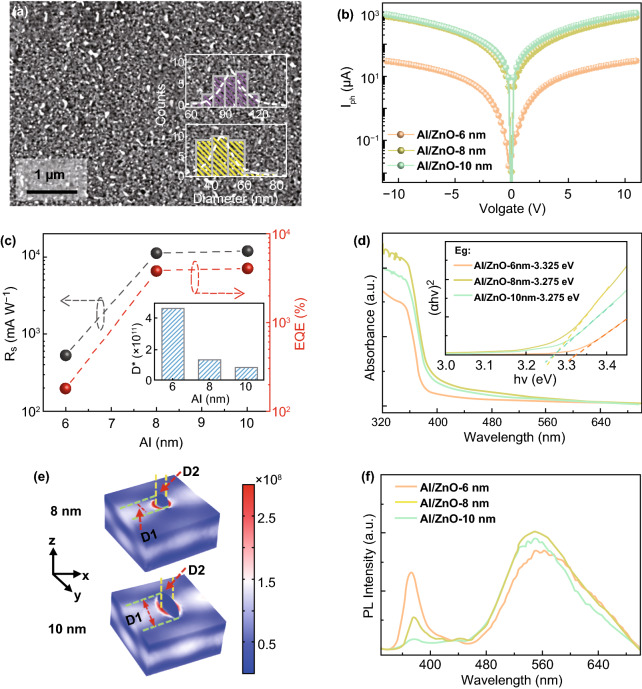
Photoresponse of the Al/ZnO heterostructure photodetectors fabricated with a variation of deposition thicknesses. **a** SEM image of the device Al/ZnO-10 nm. **b** Current–voltage curves of the devices under 365 nm light illumination (6.9 mW cm^−2^). **c** Photoresponsivity (gray) and EQE (red) of the phtodetectors fabricated with various Al deposition thicknesses. (Insets) The normalized detectivity (D*) of each device. **d** Absorbance spectra of the Al/ZnO heterostructures. (Inset) The corresponding Tauc plots. **e** The EM filed distribution of the samples Al/ZnO-8 nm and Al/ZnO-10 nm. **f** Room-temperature PL emission spectra of the samples

The morphological evolution of the self-assembled Al NSs on the ZnO QD thin films was investigated over a control of deposition thicknesses between 6 and 10 nm, and the performance of the Al/ZnO heterostructure photodetectors is shown in Fig. 5. After annealing at an identical temperature of 500 °C, the shape development of the Al NSs from tiny semi-sphere to almond-shape was witnessed with the increased deposition thicknesses as shown in Figs. 5a and S6. By providing with additional Al atoms, Al NSs tended to absorb surrounded atoms to reduce the total surface energy, and thus the size of the Al NSs radically increased at the expense of the density. As a result, at 8 nm, the average dimension of long axis (*D*1) and minor axis (*D*2) for the resulting Al NSs were ~ 78.1 and ~ 45.4 nm, respectively, accompanied with the average density of ~ 3.2 × 10^7^ (shown in Table S1). With the deposition thickness increased to 10 nm, the *D*1 and *D*2 correspondingly increased to ~ 95.6 and ~ 46.3 nm, and the density slightly decreased to ~ 2.5 × 10^7^. Meanwhile, excess Al atoms participated the inter-diffusion with the increased deposition thicknesses, leading to the increased *I*_d_ due to the improved conductivity as shown in Fig. S7. Compared with the device Al/ZnO-6 nm, the obvious enhancement in the *I*_ph_ was evidenced as a function of deposition thicknesses, and the *I*_ph_ of the device Al/ZnO-10 nm drastically increased by 36.5 times to ~ 1065.2 μA at a bias of 10 V as revealed in Fig. 5b and Table 1. Correspondingly, the *R*_*s*_ and EQE of the device Al/ZnO-10 nm increased to 11.98 A W^−1^ and 4068.7% as shown in Fig. 5c, which were apparently superior to the ZnO photodetector in previous works as summarized in Table 2. Regardless of the deposition thicknesses, each photodetector exhibited a fast response as evidenced in Fig. S8. In addition, the carrier mobility for Al/ZnO heterostructure slightly increased compared to the pristine ZnO, as shown in Fig. S9, which partially was of benefit for the improvement of photocurrent. To comprehensively evaluate the performance parameters, the device Al/ZnO-500 could be an optimal photodetector with relatively high responsivity and fast response speed.

**Table 2:** Performance comparison of the ZnO UV photodetectors with various configurations and materials

Materials	Light source	Bias (V)	*I*_ph_ (µA)	*R*_*s*_ (mA W^−1^)	*t*_rise_ (s)	*t*_decay_ (s)	EQE (%)
P–ZnO–Au [40]	245 nm	5	~ 10	–	24	15	–
Au NPs/CdMoO_4_ microplates/ZnO film [41]	350 nm, 450 W Xe lamp with a monochromator	5	0.289	321.1	16	9.2	–
Au NPs/ZnO nanowires [42]	350 nm 1.3 mW cm^−2^	1	~ 0.65	–	25	10	–
Au NPs/p-ZnO NSs/n-ZnO [43]	365 nm, 6.0 mW cm^−2^	1	–	25.4	~ 70	~ 150	8.7
Ag NWs/ZnO branched nanorods [44]	365 nm, 5 mW cm^−2^	5	250	2500	–	1	–
ZnO nanotetrapod network [45]	360 nm, 15 W cm^−2^	2.4	–	–	0.067	0.03	–
BiOCl/ZnO [10]	350 nm, 150 µW cm^−2^	5	~0.1	182.2	29.23	11.2	–
Graphene nanodot/ZnO [5]	300 nm, 403 µW cm^−2^	5	0.043	22.5	–	–	9.32
Al/ZnO-500 (this work)	365 nm, 500 µW cm^−2^	10	3.14	779.5	2.17	0.48	264.8
Al/ZnO-10 nm (this work)	365 nm, 6.9 mW cm^−2^	10	1065.2	11,976.45	6.9	19.4	4068.71

The absorbance spectra of the devices fabricated with various Al deposition thicknesses are shown in Fig. 5d. With the increased deposition thicknesses, the absorption below ~ 375 nm gradually elevated due to the enhanced LSPR, and the optical bandgap slightly decreased from ~ 3.325 to ~ 3.275 eV because of the intensified inter-diffusion of Al atoms. As shown in Fig. 5e, the size expansion of the Al NSs directly resulted in much more drastic EM fields distributed in ZnO layers, and the NBE peak correspondingly decreased due to the inhibited recombination of electron–hole as shown in Fig. 5f.

## Conclusion

In conclusion, we proposed novel Al/ZnO heterostructures for the fabrication of high-performance UV photodetectors with a comprehensive investigation of the topological effects on the photoresponse of the devices. With a variation of the annealing temperatures, the average diameter of the self-assembled Al NSs gradually evolved from ~ 48.3 to ~ 64.8 nm due to the accelerated aggregation with a broadened diffusion length of Al atoms. Depending on the deposition thicknesses, the morphological evolution of the Al NSs from the semi-spherical shape to almond shape was observed even at an identical annealing temperature. The optical properties of the Al/ZnO heterostructures sensitively developed along with the morphological evolution of the resulting Al NSs resulting from the variation of the EM fields. The photoresponse of the devices was also determined by the configuration of the heterostructures, resulting in a superior photocurrent of 1.065 mA with a photoresponsivity of 11.98 A W^−1^ under 6.9 mW cm^−2^ UV light illumination at a bias of 10 V. The relatively lower photoresponse time of ~ 0.79 s/~ 0.24 s (τ_rise_/τ_decay_) than that of the pristine ZnO device was obtained for the Al/ZnO heterostructure photodetectors, suggesting a fast response speed of the devices.

## Electronic supplementary material

Below is the link to the electronic supplementary material.

Supplementary material 1 (PDF 597 kb)
