# Supplementary material for: Self-Assembled Al Nanostructure/ZnO Quantum Dot Heterostructures for High Responsivity and Fast UV Photodetector
Source: Nanomicro Lett. 2020 May 22;12:114. doi: 10.1007/s40820-020-00455-9 (PMC7770880; doi:10.1007/s40820-020-00455-9)
Supplement: Supplementary file 1 — Supplementary material 1 (PDF 597 kb) [file 40820_2020_455_MOESM1_ESM.pdf]

Supporting Information for

# Self-Assembled Al Nanostructure/ZnO Quantum Dot Heterostructures for High Responsivity and Fast UV Photodetector

Sisi Liu[1], Ming-Yu Li[2,] *, Jianbing Zhang[1], Dong Su[1], Zhen Huang[3], Sundar Kunwar[4], Jihoon Lee[4, 5]

[1]School of Optical and Electronic Information, Huazhong University of Science and Technology, Wuhan 430074, People's Republic of China

[2]School of Science, Wuhan University of Technology, Wuhan 430070, People's Republic of China

[3]Wuhan National Laboratory for Optoelectronics (WNLO) and School of Engineering Sciences, Huazhong University of Science and Technology, Wuhan 430074, People's Republic of China

[4]College of Electonics and Information, Kwangwoon University, Nowon-gu Seoul 01897 Republic of Korea

[5]Institute of Nanoscale Science and Engineering, University of Arkansas, Fayetteville, AR 72701, USA

*Corresponding author. E-mail: mingyuli.oliver@gmail.com (Ming-Yu Li)

## Supplementary Figures and Table

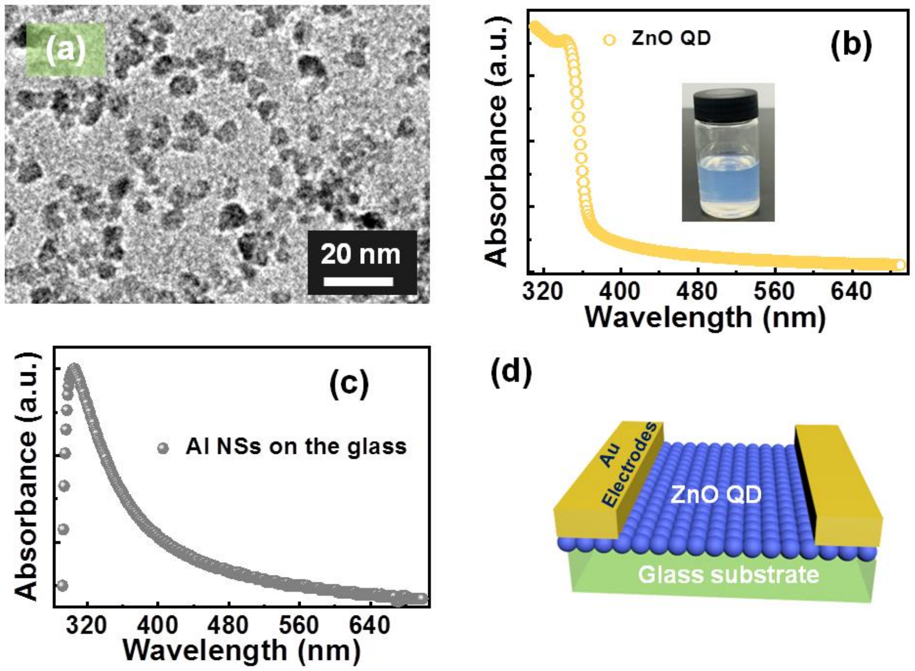


**Fig. S1** (**a**) Transmission electron microscope image of ZnO quantum dots (QD). (**b**) Absorbance spectra and (Insets) optical image of ZnO QD solution. (**c**) Absorbance spectra of the Al NSs on the glass substrate. (**d**) The structure of the pristine ZnO device

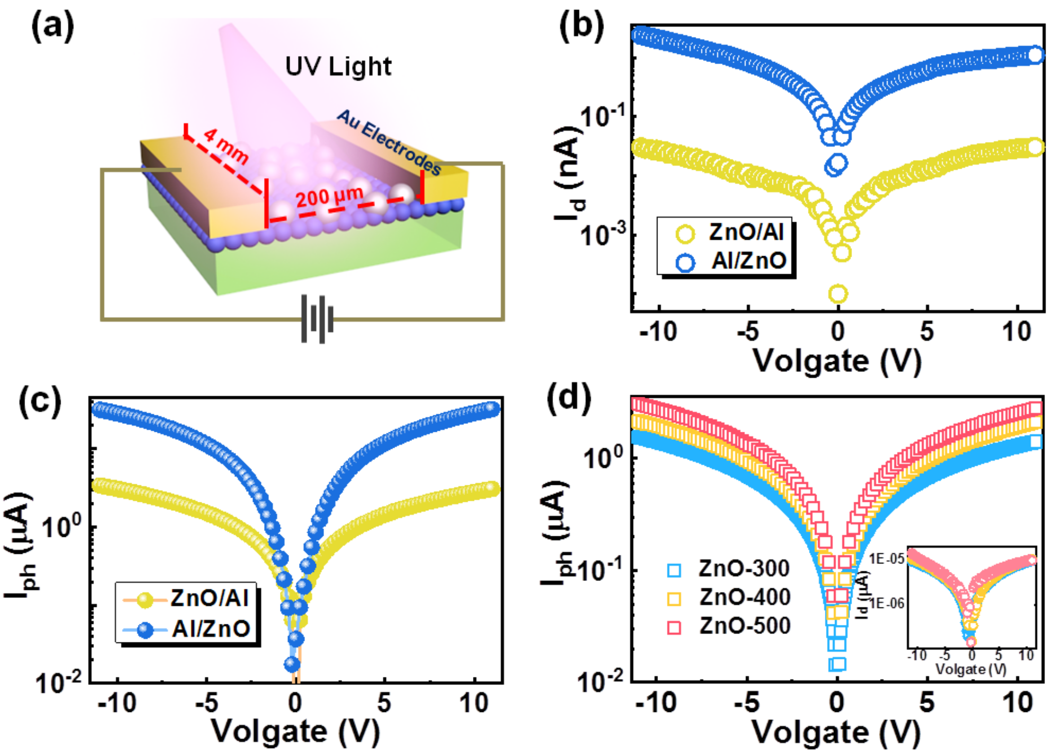


**Fig. S2** (**a**) The schematic diagram of the configuration of the Al/ZnO heterostructure photodetector. Current-voltage curves of the photodetectors with various configurations (**b**) in the dark and (**c**) under 365 nm light illumination (6.9 mW $cm^{-2}$). (**d**) Current-voltage curves of the ZnO photodetectors annealed at various temperature under 365 nm light illumination (6.9 mW $cm^{-2}$) and in the dark (inset)

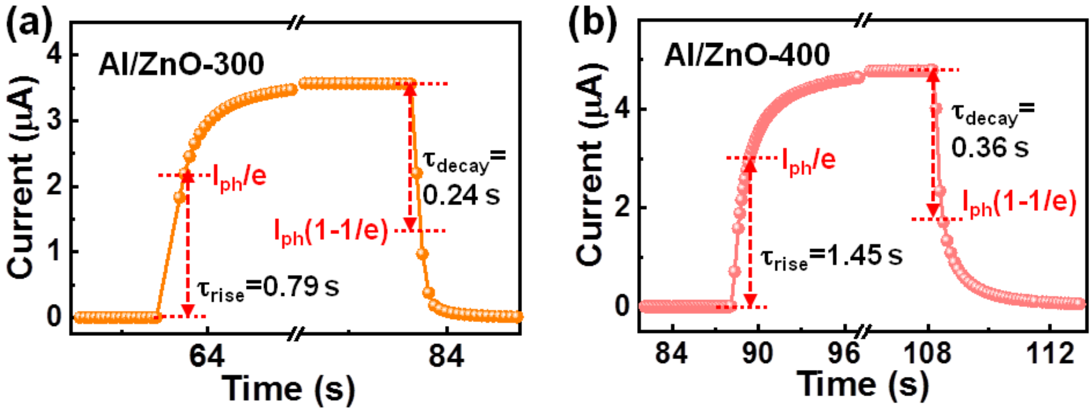


**Fig. S3** Time-resolved photoresponse of the Al/ZnO photodetectors fabricated at different annealing temperatures under 365 nm light illumination (6.9 mW $cm^{-2}$) at a 10 V bias: (**a**) 300 °C and (**b**) 400 °C.

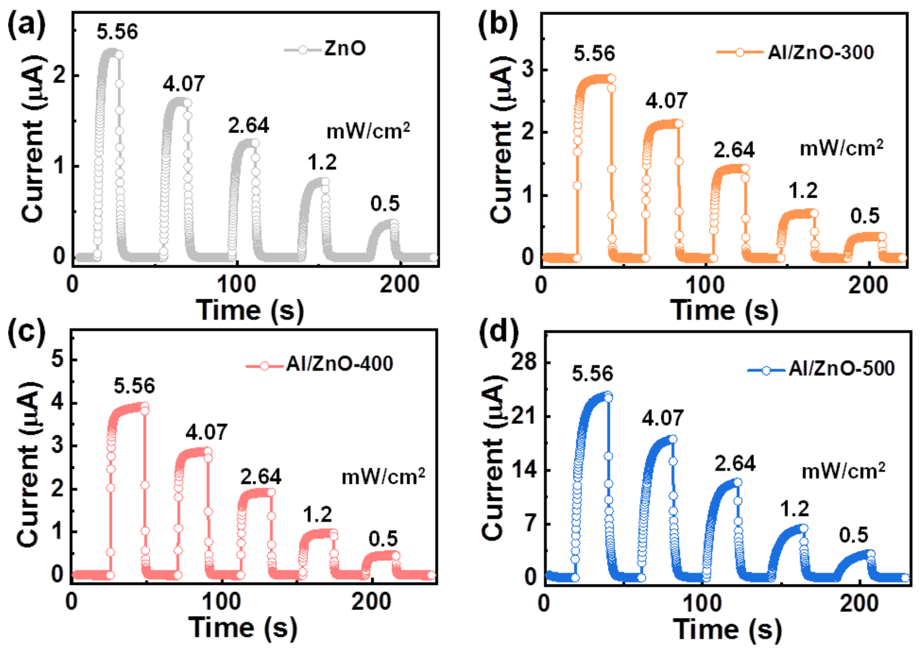


**Fig. S4** Light intensity dependent photoresponse of the pristine ZnO (**a**) and Al/ZnO heterostructure detectors fabricated at different temperatures under 365 nm light illumination at a 10 V bias: (**b**) 300, (**c**) 400, and (**d**) 500 °C

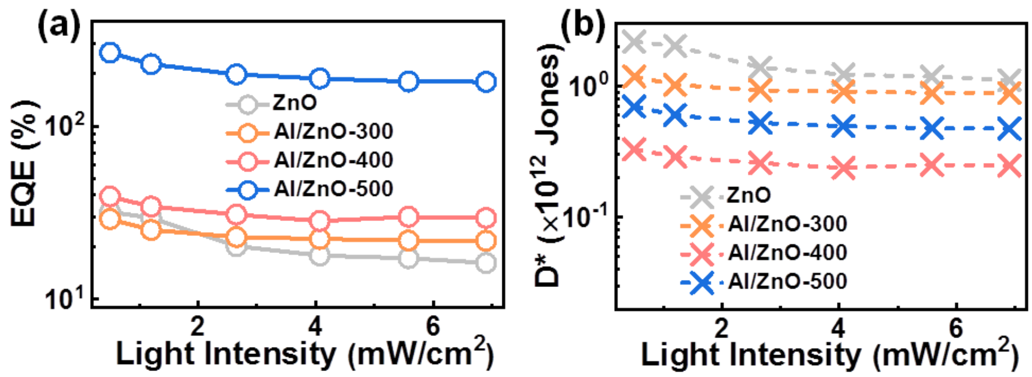


**Fig. S5** (**a**) EQE and (**b**) *D** of each device under 365 nm UV illumination at various light intensities

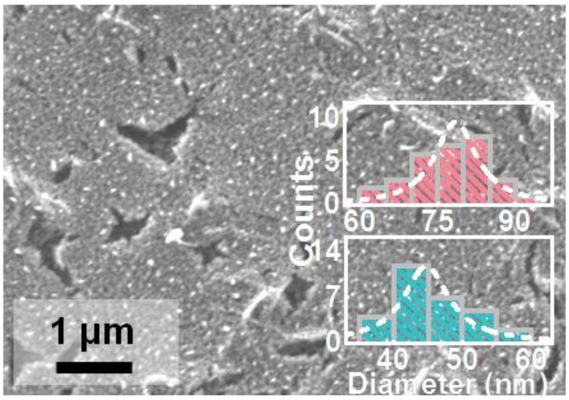


**Fig. S6** SEM images of the device Al/ZnO-8 nm

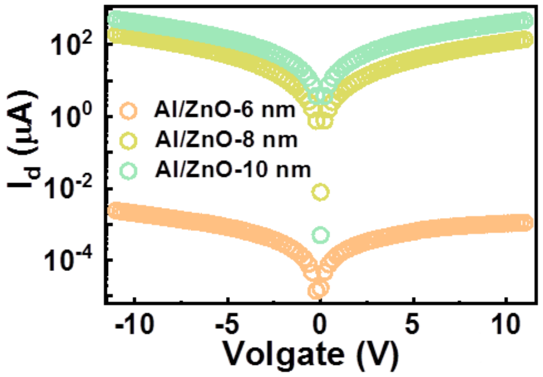


**Fig. S7** Current-voltage curves of the device fabricated with various deposition thicknesses

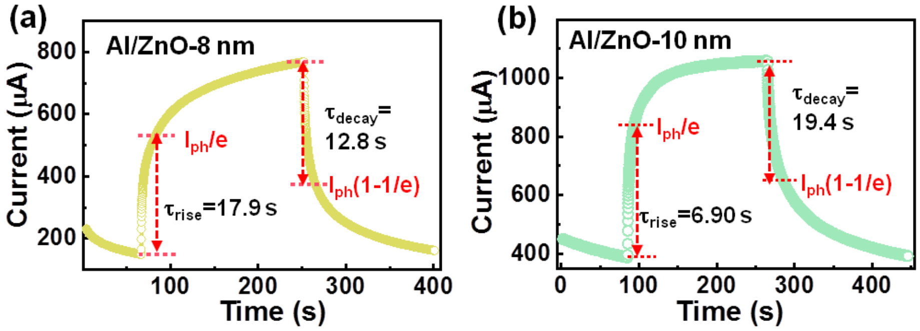


**Fig. S8** Time-resolved photoresponse of devices (**a**) Al/ZnO-8 nm and (**b**) Al/ZnO-10 nm under 365 nm light illumination (6.9 mW $cm^{-2}$) at a 10 V bias

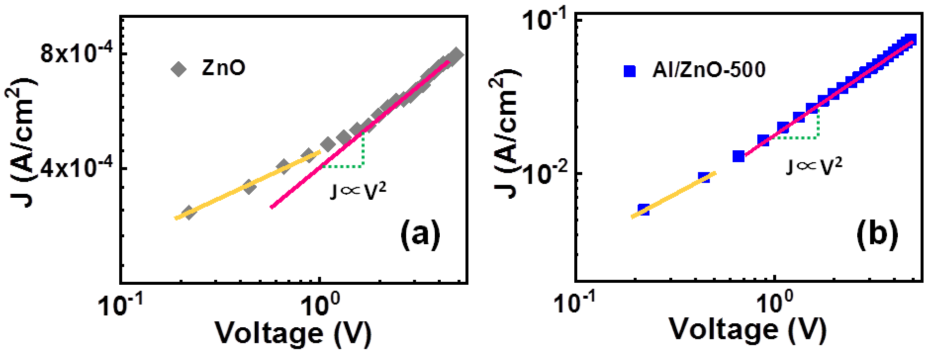


**Fig. S9** Logarithmic current density-voltage (J-V) characteristic of the pristine ZnO (**a**) and Al/ZnO-500 heterostructure (**b**)

**Table S1** Average dimension of long axis (*D*1) and minor axis (*D*2), and density of the Al NSs of the devices Al/ZnO-8 nm and Al/ZnO-10 nm

| Samples | *D1* (nm) | *D2* (nm) | Density ($cm^{-2}$) |
|---|---|---|---|
| Al/ZnO-8 nm | 78.1 | 45.4 | $3.2\times10^{7}$ |
| Al/ZnO-10 nm | 95.6 | 46.3 | $2.5\times10^{7}$ |

## S2 Caculation of the Carrier Mobility

The electron mobility was calculated by using the Mott–Gurney space-charge-limitedcurrent (SCLC) equation [S1, S2]:

$$J = \frac{9}{8}\mu_{eff}\varepsilon_0\varepsilon_r\frac{V^2}{d^3}$$

where $J$ is the current density, $\mu_{eff}$ is the effective charge carrier mobility, $\varepsilon_0$ is the permittivity of the vacuum, $\varepsilon_r$ is the relative dielectric constant, $V$ is the applied voltage, and $d$ is the thickness of the active layer. The value of $\varepsilon_r$ for ZnO is 2.9 according to the previous reports [S3]. The effective carrier mobilities are 0.084 and 0.197 cm$^2$ V$^{-1}$ s$^{-1}$ for the pristine ZnO and Al/ZnO heterostructure, respectively. Thus, the inter-diffusion of Al to ZnO matrixs slightly improved the carrier mobility, which was of benefit for the improvement of photocurrent.

## Supplementary References


[S1] C. Bi, S.V. Kershaw, A.L. Rogach, J. Tian, Improved stability and photodetector performance of $CsPbI_3$ perovskite quantum dots by ligand exchange with aminoethanethiol, Adv. Funct. Mater. **29**, 1902446 (2019) https://doi.org/10.1002/adfm.201902446

[S2] J.S. Park, J.M. Lee, S.K. Hwang, S.H. Lee, H.-J. Lee, B.R. Lee, H.I. Park, J.-S. Kim, S. Yoo, M.H. Song, S.O. Kim, A ZnO/N-doped carbon nanotube nanocomposite charge transport layer for high performance optoelectronics, J. Mater. Chem. **22**, 12695-12700 (2012). https://doi.org/10.1039/C2JM30710C

[S3] S. Middya, A. Layek, A. Dey, J. Datta, M. Das, C. Banerjee, P.P. Ray, Role of zinc oxide nanomorphology on Schottky diode properties, Chem. Phys. Lett. **610**(611), 39-44 (2014). http://dx.doi.org/10.1016/j.cplett.2014.07.003
